# The impact of good governance on organizational health with the mediating role of organizational accountability: considering the influence of demographic and organizational variables

**DOI:** 10.1186/s12913-025-12921-4

**Published:** 2025-07-02

**Authors:** Taiebeh Taheri, Mohammad Amerzadeh, Ahad Alizadeh, Rohollah Kalhor

**Affiliations:** 1 Student Research Committee, School of Public Health, Qazvin University of Medical Sciences, Qazvin, Iran; 2 Non-communicable Diseases Research Center, Research Institute for Prevention of Non- Communicable Diseases, Qazvin University of Medical Sciences, Qazvin, Iran

**Keywords:** Good governance, Organizational health, Organizational accountability, Academic hospitals

## Abstract

**Background:**

Human resources are the critical element of organizations and serve as the economic engine of countries. Human resources play a crucial role in gaining competitive advantage. This study aimed to examine the impact of good governance on organizational health, with organizational accountability as a mediating factor, among employees of hospitals in Qazvin, Iran.

**Methods:**

This analytical, applied research used a descriptive design and was conducted in selected hospitals in Qazvin in 2023. Data were collected from hospital staff who evaluated their hospitals’ organizational attributes, including good governance, organizational health, and accountability. Descriptive and inferential statistics were used to analyze the data. Standard questionnaires, including Singh and Jha’s (2018) Organizational Health, Moghimi and Ramazan’s (2011) Good Governance, and Jansen (2001) Organizational Accountability were used for data collection. To analyze the data and test the hypotheses, Pearson correlation coefficients were employed. Structural Equation Modeling (SEM) was employed for data analysis.

**Results:**

The findings indicated that good governance directly affects organizational health and organizational accountability. Scores for good governance, organizational health, and organizational accountability in Qazvin hospitals were satisfactory. Good governance scores were higher among personnel with fixed shifts, contract staff, those with high school diplomas or lower education, educational groups and married personnel.

**Conclusion:**

Based on the study results, hospital managers can enhance organizational health by improving governance and accountability. Further nationwide studies on the impact of organizational accountability on organizational health are recommended. To further advance the understanding of organizational health, future research should explore several key avenues.

## Background

Human resources are the most critical component of organizations, and they serve as the economic engines of nations. Human resources play a vital role in gaining competitive advantage [1]. They are considered the economic gears of any country; the quality, competence, capability, and ability of the human resources at an organization’s disposal are decisive in determining the level of success and achieving strategic and dynamic competitive advantage for organizations in today’s dynamic environment [2].

Organizations, akin to living organisms, obtain the materials and energy necessary for survival from their surrounding environment and, in turn, provide services or products to the environment. Therefore, if an organization is unhealthy, it leads to the illness of its members and society. Having a healthy society is inconceivable without healthy organizations. The pulse of social health depends on the health of organizations. Several studies have been conducted on organizational health, some related to education and organizational health, while others have shifted toward administrative corruption [3].

Organizations must adopt structural and content-based measures appropriate to their conditions to prevent the consequences of weak organizational health. One of these measures is undoubtedly institutionalizing good governance in organizations. Governance is a set of individual and institutional, public and private actions for planning joint administration of affairs and a continuous process of creating understanding between different and conflicting interests. It takes place in the form of participatory and compatible actions and includes formal institutions, informal arrangements, and social capital of citizens [4].

Good governance has been introduced as a creator of mutual, supportive, and collaborative relationships between the government, civil society, and the private sector. The nature of relationships between these three active groups and the necessity of powerful mechanisms to facilitate interaction between them are fundamental assumptions. Good governance is a concept that encompasses components such as participation, transparency, accountability, rule of law, foresight ability, democratic practices, and civil liberties [5].

Accountability is one of the primary objectives and components considered by policymakers and health systems managers. All health systems worldwide are seeking ways to be more responsive to patients and the communities. The World Health Organization (WHO) framework for evaluating health system performance revolves around health, responsiveness, and financial fairness as the primary objectives of any health system. Today, accountability has gained increasing importance and is a systemic concept: outcomes or benefits for customers achieved when organizational and external relationships are designed to provide adequate recognition and appropriate response to customers’ reasonable expectations [6].

Accountability includes two components: respect for individuals, which encompasses dignity, confidentiality, and autonomy for individuals and families to make decisions about their health; and customer orientation, which encompasses prompt attention, access to social support networks in care, maintenance, quality of basic welfare facilities, and choice of service provider or relevant therapist. Accountability is not a measure of how individuals’ health needs met or clinical outcomes are, but rather how a system responds to non-health aspects, whether individuals’ expectations are met or not, and how service providers interact with them [7].

Therefore, the importance of accountability and organizational health in hospitals for fulfilling their mission led us to seek a solution to improve organizational health in hospitals. Since good governance and organizational accountability are factors that positively affect organizational health, and given the lack of research on good governance and accountability in hospitals in Qazvin, this study aimed to examine the effect of good governance with the mediating role of accountability on organizational health. By exploring the relationships between good governance, organizational accountability, and organizational health, this study aims to provide insights that can help improve the functioning and effectiveness of academic hospitals in Qazvin. The findings may have implications for hospital management practices and policies, potentially leading to enhanced healthcare delivery and patient satisfaction.

In recent years, governance has become a critical issue in the management of the public and government sectors, owing to the significant role it plays in shaping organizational health. Key pillars of good governance include transparency, responsibility, accountability, participation, rule of law, and governmental flexibility. A healthy organization is one where individuals want to stay, work, and be both productive and effective [8]. Therefore, a healthy organization fosters the concept of organizational health, motivating and engaging employees in their work. Accountability is regarded as a strategic tool through which organizational performance is assessed, customer satisfaction is enhanced, and operational efficiency and business process improvement are achieved. Accountability is a particularly important issue for health policymakers and managers.

Accountability can be considered one of the most crucial components of good governance. Alongside governmental institutions, private organizations and civil society actors must also be accountable for their policies and actions. It should be noted that the principles of good governance are interconnected, with the implementation of each principle depending on the execution of others. For instance, it is unrealistic to expect accountability and responsibility to be effectively operational without the presence of transparency and the rule of law.

Hospitals are the largest and most expensive operational units within the healthcare system, consuming a significant portion of financial and human resources. As organizations that play a critical role in the nation’s health, improving the performance of hospitals directly contributes to the advancement of public health. Therefore, identifying the prerequisites for improving hospital performance is a crucial step toward enhancing their effectiveness. According to the literature and previous research in this field, accountability and organizational health not only improve organizational performance but also enable hospitals to effectively fulfill their role in advancing the nation’s healthcare development.

Moreover, several demographic and organizational factors—such as shift type, organizational field, employment type, educational level, marital status, gender, and workplace—may influence perceptions and behaviors related to good governance, accountability, and organizational health. Recognizing the potential role of these variables, this study considers them in its design to better understand whether and how individual and contextual characteristics contribute to the dynamics of hospital governance and performance. These variables are included in the descriptive analysis and considered for their potential moderating or confounding effects, even though they are not central to the structural equation model (SEM) used in the hypothesis testing.

Based on a review of theoretical texts and studies conducted both domestically and internationally, it has been observed that there is a limited amount of research on good governance among employees of medical universities, despite the recognized importance of the topics of good governance, organizational health, and organizational accountability. Furthermore, in the healthcare sector, there is a notable scarcity of studies examining the interrelationship among the three variables: good governance, organizational health, and organizational accountability.

Despite existing studies on the impact of good governance on organizational health, there has yet to be a comprehensive and simultaneous examination of the influence of good governance on organizational health with the mediating role of organizational accountability in educational and healthcare centers in Qazvin. The aim of this study is to investigate the effect of good governance on organizational health, considering the mediating role of organizational accountability among the staff of university hospitals in Qazvin.

In the hospitals of Qazvin, as important governmental centers, managers must implement policies that reflect the principles of good governance aimed at enhancing organizational transparency, increasing incentives for innovation, and boosting employee morale, particularly given their challenging and sensitive roles. This, in turn, should lead to improved organizational health. Hospitals should create conditions that allow feedback and suggestions from the public and stakeholders regarding the quality of services to reach the relevant authorities. Administrative corruption, which stands in stark contrast to administrative health, poses a significant challenge in public organizations. The model of good governance is proposed as a solution to combat administrative corruption and establish organizational health.

The novelty of this study lies in several key aspects. First, while previous studies have examined these variables separately or in pairs, this is the first comprehensive study to investigate the three-way relationship between good governance, organizational health, and organizational accountability in healthcare settings. Second, this research is pioneering in exploring these relationships specifically within Iranian academic hospitals, providing valuable insights into the healthcare sector of a developing country. Third, the study’s unique contribution is its examination of organizational accountability as a mediating factor between good governance and organizational health - a relationship that has not been previously explored in healthcare organizations. Finally, this research provides a novel framework for understanding how good governance practices can be effectively implemented in healthcare settings through the mechanism of organizational accountability to achieve better organizational health outcomes. These distinctive features make this study particularly relevant for both academic research and practical healthcare management.

Based on the theoretical framework and research objectives, this study tested the following hypotheses:

1. Good governance has a significant direct effect on organizational health in Qazvin hospitals.
2. Good governance has a significant direct effect on organizational accountability in Qazvin hospitals.
3. Organizational accountability has a significant direct effect on organizational health in Qazvin hospitals.
4. Good governance has a significant indirect effect on organizational health through the mediating role of organizational accountability in Qazvin hospitals.

These hypotheses were formulated to examine both the direct relationships between the variables and the mediating role of organizational accountability in the relationship between good governance and organizational health. The hypotheses were designed to provide a comprehensive understanding of how these organizational factors interact within the hospital setting and to guide the statistical analysis of the collected data. Testing these hypotheses would help validate the theoretical framework and provide practical insights for hospital management.”

## Methods

### Study design

This analytical, applied research employed a descriptive approach and was conducted in selected hospitals in Qazvin in 2023. Data were collected from hospital staff who evaluated their hospitals’ organizational attributes, including good governance, organizational health, and accountability. Descriptive and inferential statistics were utilized for data analysis.

To gather data, the first step involved identifying the number of hospitals affiliated with Qazvin University of Medical Sciences. Subsequently, the number of staff working in these centers was determined, which allowed for the specification of the sample size. After providing an explanation regarding the research objectives, staff were asked to complete the relevant questionnaires. The distribution of the questionnaires to the staff took place over a period of three months. Additionally, during this process, the participating staff were requested to ensure that all information recorded in the questionnaires was completed accurately and thoroughly.

The primary technique employed for data analysis in this study is SEM. This method is one of the most robust and suitable approaches for multivariate analysis in behavioral and social science research. The nature of such topics is inherently multivariate, making it inappropriate to analyze them using bivariate methods, which consider only one independent variable and one dependent variable at a time.

Multivariate analysis refers to a set of analytical methods characterized by the simultaneous examination of K independent variables and N dependent variables. Covariance structure analysis, causal modeling, or structural equation modeling is one of the fundamental methods for analyzing complex data structures. Therefore, since this study involves multiple independent variables that need to be assessed for their effects on the dependent variable, the use of SEM is deemed essential.

To analyze the data and test the hypotheses, Pearson correlation coefficients were employed.

### Data collection

The primary study population comprised all employees (clinical staff, administrative staff, financial personnel, and paraclinical staff) of the educational hospitals affiliated with Qazvin University of Medical Sciences (QUMS), located in Qazvin City. This included six hospitals: Rajaee, Kosar, Booali, Qods, Velayat, and 22 Bahman.

The total number of employees in these educational-medical centers was 2,256. Based on Morgan’s table, the required sample size was determined to be 331. Accounting for a 10% sample attrition rate, 365 questionnaires were distributed proportionally according to the number of staff in each hospital.

Stratified sampling was used to determine the number of individuals from each stratum (hospital). Within each stratum, systematic random sampling was employed to select the required sample.

The sample distribution across hospitals was as follows: Rajaee Hospital 68 samples, Kosar Hospital 57 samples, Booali Hospital 92 samples, Qods Hospital 52 samples, Velayat Hospital 77 samples and 22 Bahman Hospital 19 samples.

The data collection tool consisted of 4 sections. The first section included demographic information (age, gender, education, work experience, organizational position, etc.) The second to fourth sections included the standardized organizational health questionnaire by Singh and Jha [9], the good governance questionnaire by Moghimi and Ramezani [10], and the organizational accountability questionnaire by Jansen [11], respectively.

The standardized organizational health questionnaire by Singh and Jha [9] with 19 items and seven components (managerial effectiveness, pleasant power relations, human resource development, team orientation, organizational values, innovation, and morale) was used to confirm the validity of the measurement tool. Three types of validity were used: content validity (confirmed through expert opinion), convergent validity (measured using the average variance extracted), and discriminant validity (measured using the Fornell-Larcker method). Two criteria were used to determine the reliability of the questionnaire: Cronbach’s alpha coefficient and composite reliability coefficient.

The good governance questionnaire [10] consists of 35 items and six components (result orientation, effectiveness of roles and responsibilities, promotion of values, transparency, capacity building, and accountability). The reliability of the questionnaire items was calculated using Cronbach’s alpha method, which was 0.723 for the good governance variable. The validity of the questionnaire items was confirmed through content validity and expert opinion.

The organizational accountability questionnaire [11] (1) consists of 15 items and includes legal accountability ،financial accountability, ethical accountability، functional accountability, and democratic accountability. Three types of validity were used to confirm the validity of the measurement tool: content validity (confirmed through expert opinion), convergent validity (measured using the average variance extracted), and discriminant validity (measured using the Fornell-Larcker method). Two criteria were used to determine the reliability of the questionnaire: Cronbach’s alpha coefficient and composite reliability coefficient.

All the questionnaire items were closed-ended and scored on a five-point Likert scale, where each response was assigned a score from 1 to 5. This indicates an acceptable face and content validity of the data collection tool.

### Data analysis

Descriptive and inferential statistics were used for data analysis. In addition, the research hypotheses were analyzed at the 0.05 error level using the AMOS software.

The primary technique used for data analysis in this research is SEM. One of the most robust and appropriate methods of analysis in behavioral and social science research is multivariate analysis. This is because the nature of such subjects is multivariate, and they cannot be analyzed using bivariate methods. Multivariate analysis refers to a series of analytical methods whose main characteristic is the simultaneous analysis of K-independent and N-dependent variables.

Analysis of covariance structures, causal modeling, or SEM is one of the primary methods for analyzing complex data structures. Therefore, since this study involves several independent variables whose effects on the dependent variable need to be examined, SEM becomes necessary.

Pearson’s correlation coefficient was used to analyze the information and test hypotheses. Two statistical software packages were employed to examine the results: SPSS23 and AMOS. Using both SPSS and AMOS software ensures a comprehensive data analysis, with SPSS facilitating descriptive statistics and preliminary analyses. AMOS also enables the construction and testing of the SEM. This dual approach to data analysis enhances the robustness and reliability of the research findings.

## Results

The results of the descriptive analysis showed that out of 330 participants, 93 (18.28%) were male and 237 (82.71%) were female. Among the respondents, 31.52% (104 individuals) were single, while 68.48% (226 individuals) were married. Besides, 5.45% had an associate degree or lower, 68.48% a bachelor’s degree, 18.79% a master’s degree, and 7.27% a PhD or higher. The mean age of the participants was 36.742 ± 7.532 years. The mean work experience of the participants was 11.500 ± 7.467 years. The mean managerial experience was 0.697 ± 2.738 years (Table 1).

**Table 1:** Demographic information of participants

	MeanSD	MeanSE	CI	MedianQuantile
Age	36.742 ± 7.532	36.742 ± 0.415	36.742 (35.930, 37.555)	36.000 (30.000, 42.000)
Work Experience	11.500 ± 7.467	11.500 ± 0.411	11.500 (10.694, 12.306)	11.000 (5.000, 16.000)
Management experience	0.697 ± 2.738	0.697 ± 0.151	0.697 (0.402, 0.992)	0.000 (0.000, 0.000)

The overall mean score for good governance was 108.54 ± 22.14 (*P*-value = 0.008), for organizational health 56.16 ± 12.52 (*p*-value = 0.04), and for organizational accountability 47.74 ± 8.72 (*p*-value = 0).

Based on t-test analysis for the scores of each questionnaire according to the type of work shift, the organizational health score for employees with fixed shifts was 57.51 ± 13.16, which was 2.73 points higher than the score for personnel with rotating shifts (*P*-value = 0.04) (Table 2). Additionally, the good governance score for fixed-shift employees was assessed at 111.73 ± 23.53, 6.47 points higher than personnel with rotating shifts. Furthermore, the organizational accountability score for personnel with fixed shifts was 49.43 ± 8.70, and for personnel with rotating shifts was 46.01 ± 8.42 (Table 3).

**Table 2:** Results of analysis of variance (ANOVA) based on educational level

Factor	Associate’s degree and below	Bachelor’s degree	Master’s degree	PhD and above	*P* value
Age	44.33 ± 4.27	35.81 ± 7.32	39.44 ± 7.82	32.83 ± 4.80	< 0.001
Work_Experience	17.33 ± 6.82	11.00 ± 7.21	13.89 ± 7.44	5.62 ± 5.21	< 0.001
Organizational_Health	59.56 ± 16.03	55.53 ± 12.15	57.50 ± 13.41	55.79 ± 10.09	0.452
Good governance	113.56 ± 26.57	107.73 ± 21.71	111.52 ± 22.66	103.58 ± 20.25	0.314
Organizational_Accountability	50.67 ± 8.64	47.30 ± 8.89	49.16 ± 8.35	46.00 ± 7.25	0.156

**Table 3:** Results of T-test analysis based on shift type

Factor	Overall	Fixed shift	Rotating shift	*P*-value
Age; Mean ± SD	36.77 ± 7.53	39.33 ± 7.25	34.14 ± 6.90	0
Work Experience: Mean ± SD	11.52 ± 7.47	13.72 ± 7.55	9.25 ± 6.70	0
Organizational Health; Mean ± SD	56.16 ± 12.52	57.51 ± 13.16	54.78 ± 11.69	0.047
Good governance; Mean ± SD	108.54 ± 22.14	111.73 ± 23.53	105.26 ± 20.16	0.008
Organizational Accountability; Mean ± SD	47.74 ± 8.72	49.43 ± 8.70	46.01 ± 8.42	0

The results of the analysis of variance (ANOVA) showed that the organizational health score was higher in the educational group than other occupational groups, and lower in the healthcare and administrative-financial groups than other groups (*p*-value = 0.09) (Table 4). Similarly, good governance also had the highest score in the educational group and the lowest score in the healthcare group (*p*-value = 0.2) (Table 4). Organizational accountability was also higher in the educational group than other groups and lower in the healthcare group than other groups (*p*-value = 0.03) (Table 4).

**Table 4:** Results of analysis of variance (ANOVA) based on occupational field

Factor	Healthcare	Administrative and Financial	Technical and Engineering	Educational	*P* value
Age	35.18 ± 7.21	40.57 ± 6.72	38.57 ± 6.95	46.25 ± 9.43	< 0.001
Work_Experience	10.27 ± 7.11a	14.98 ± 7.33	10.00 ± 5.48	14.50 ± 12.40	< 0.001
Organizational_Health	55.97 ± 11.57	55.41 ± 14.67	63.71 ± 8.06	67.75 ± 16.15	0.095
Good governance	107.91 ± 20.71	108.33 ± 26.33	120.43 ± 9.98	122.75 ± 13.72	0.276
Organizational_Accountability	47.05 ± 8.57	48.93 ± 8.97	52.14 ± 3.63	56.25 ± 10.24	0.033

As illustrated in Table 5, the scores for good governance, organizational accountability, and organizational health were higher for contract personnel than for other employment types. Conversely, project-based personnel exhibited the lowest scores in these areas (see Table 5).

**Table 5:** Results of analysis of variance (ANOVA) based on employment type

Factor	Formal	Contractual	Project-based/Contract-based	Company-based	*P* value
Age	39.28 ± 6.86	34.78 ± 6.22	34.12 ± 7.83	33.75 ± 5.61	< 0.001
Work_Experience	14.11 ± 7.06	9.47 ± 5.56	8.75 ± 7.45	9.00 ± 5.95	< 0.001
Organizational_Health	57.36 ± 13.06	54.39 ± 9.25	54.53 ± 11.99	60.58 ± 15.88	0.123
Good governance	111.10 ± 22.7	104.00 ± 19.01	105.73 ± 21.40	112.50 ± 24.88	0.113
Organizational_Accountability	48.98 ± 8.80	46.11 ± 7.49	46.47 ± 8.62	48.33 ± 9.99	0.068

To compare the means of the main variables of the study based on employment status, a one-way analysis of variance (ANOVA) was employed. According to Table 5, good governance and organizational health were higher among employees with other employment statuses compared to other personnel. Specifically, good governance was lower in contract employees than in other groups, while organizational health was also observed to be lower among project-based contract employees relative to their counterparts. Organizational accountability was higher in permanent staff compared to other employees, whereas contract employees exhibited lower levels than the others. However, no significant differences were found between the mean scores of employment status and the study variables.

Based on the analysis of variance (ANOVA) with personnel’s educational level, the scores for all questionnaires were higher for personnel with a diploma or lower education than other educational levels (Table 2). Additionally, based on the independent t-test results, questionnaire scores were higher for married individuals than single individuals (Table 6).

**Table 6:** Results of t-test analysis based on marital status

Factor	Total	Married	Single	*P*- value
Age; Mean ± SD	36.74 ± 7.53	39.32 ± 6.64	31.14 ± 6.21	0
Work_Experience; Mean ± SD	11.50 ± 7.47	13.86 ± 7.03	6.38 ± 5.61	0
Organizational_Health; Mean ± SD	56.14 ± 12.48	56.30 ± 12.83	55.79 ± 11.74	0.721
Good governance; Mean ± SD	108.46 ± 22.09	109.93 ± 23.41	105.25 ± 18.60	0.053
Organizational_Accountability; Mean ± SD	47.74 ± 8.70	48.35 ± 8.66	46.42 ± 8.67	0.063

The results of the independent t-test based on gender showed that questionnaire scores were approximately the same for both genders, with the difference that the good governance score was evaluated higher in women than men (*P*-value = 0.3) (Table 7). However, the situation regarding organizational accountability is different, as male employees exhibited higher levels than their female counterparts. Nonetheless, no significant differences were found between the mean scores of good governance, organizational health, and organizational accountability based on the participants’ gender.

**Table 7:** Results of t-test analysis based on gender

Factor	Total	Male	Female	*p*-Value
Age; Mean ± SD	36.74 ± 7.53	36.83 ± 7.25	36.71 ± 7.66	0.895
Work_Experience; Mean ± SD	11.50 ± 7.47	11.69 ± 7.55	11.43 ± 7.45	0.776
Organizational_Health; Mean ± SD	56.14 ± 12.48	56.00 ± 11.07	56.19 ± 13.02	0.892
Good governance; Mean ± SD	108.46 ± 22.09	106.49 ± 22.25	109.23 ± 22.03	0.317
Organizational_Accountability; Mean ± SD	47.74 ± 8.70	47.97 ± 8.08	47.65 ± 8.95	0.758

Based on the ANOVA about the hospitals, the score of good governance in Velayat Hospital was evaluated as higher than other hospitals (*p*-value=0.28). The score of organizational health was also higher in Velayat Hospital than other hospitals (*p*-value=0.39). The score of organizational accountability was also evaluated higher in Velayat Hospital than other hospitals (*p*-value=0.10) (Table 8).

**Table 8:** Results of analysis of variance (ANOVA) based on workplace

Factor	Rajaee	Kasar	Buali	Quds	Velayat	22 Bahman	*P* value
Age	36.12 ± 8.93	36.47 ± 7.22	35.97 ± 7.66	37.76 ± 7.87	37.21 ± 5.91	38.19 ± 8.45	0.692
Work_Experience	11.40 ± 8.76	12.00 ± 7.08	11.07 ± 7.90	11.36 ± 7.75	11.32 ± 5.77	12.67 ± 8.55	0.956
Organizational_Health	55.67 ± 9.97	54.18 ± 14.60	55.14 ± 11.84	56.26 ± 13.21	58.44 ± 12.98	58.14 ± 10.78	0.399
Good governance	109.36 ± 16.35	106.31 ± 25.77	104.14 ± 22.61	109.55 ± 20.02	112.72 ± 23.98	109.19 ± 18.62	0.282
Organizational_Accountability	46.80 ± 6.81	46.50 ± 10.25	46.54 ± 8.76	49.50 ± 8.15	49.76 ± 9.06	47.35 ± 6.92	0.104

**Fig. 1:**
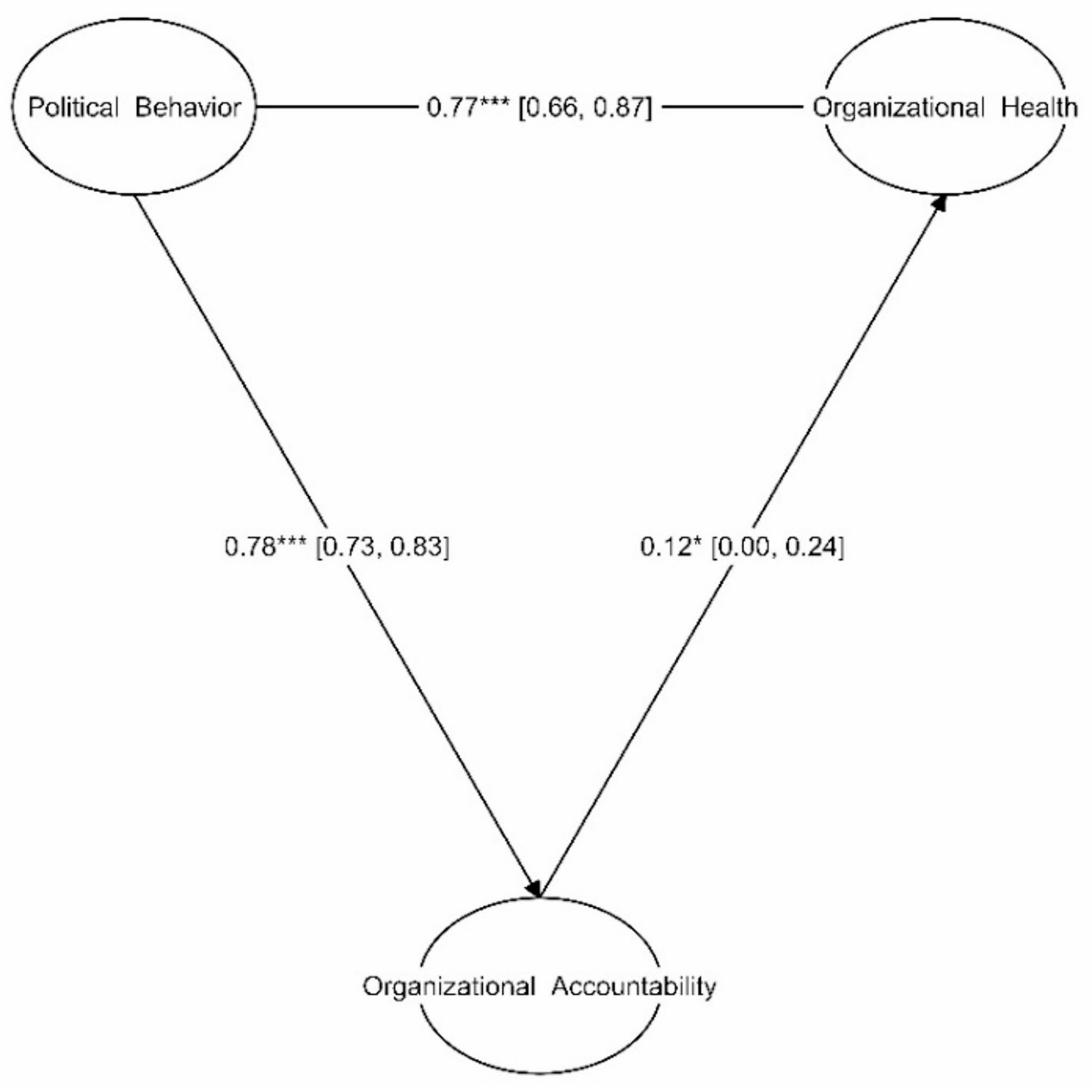
Estimation of the research model with standardized coefficients

Figure 1 illustrates the estimation of the research model using standardized coefficients.

Table 9 examines the relationship between each of the items of age, work experience, and the three questionnaires evaluated. As shown, all items have a direct relationship with each other, and based on the evaluated *p*-values, all data were statistically significant.

**Table 9:** Correlation between different factors

	Age	Work Experience	Organizational_Health	Political_Behavior	Organizational_Accountability
Age	1.00 (NA)	0.90(0.000)	0.10 (0.062)	0.14 (0.010)	0.15 (0.006)
Work Experience	0.90(0.000)	1.00 (NA)	0.08 (0.147)	0.11 (0.057)	0.11 (0.042)
Organizational_Health	0.10(0.062)	0.08(0.147)	1.00 (NA)	0.81 (0.000)	0.65 (0.000)
Good governance	0.14(0.010)	0.11(0.057)	0.81 (0.000)	1.00 (NA)	0.74 (0.000)
Organizational_Accountability	0.15(0.006)	0.11(0.042)	0.65 (0.000)	0.74 (0.000)	1.00 (NA)

To fit the model, the SEM method was employed, resulting in the following findings in Table 10:

**Table 10:** Fit indices for the refined model

Index Name	Acceptable Limit	Obtained Value
RMSEA	Less than 0.08	0.072
IFI	Greater than 0.90	0.736
CFI	Greater than 0.90	0.734
GFI	Greater than 0.90	0.605
PNFI	Greater than 0.50	0.618

Based on the data analysis presented in Table 11, good governance demonstrates a significant direct and total relationship with organizational health. The indirect relationship is marginally significant (*p* < 0.10). Overall, this suggests that Political Behavior has a strong positive influence on Organizational Health, both directly and (to a lesser extent) indirectly through Organizational Accountability.

**Table 11:** Main research hypothesis results (95% confidence interval)

Effects	Unstandardized Coefficient	Standardized Coefficient	95% Confidence Interval		z-value	*P*-value
			Lower Bound	Upper Bound		
Organizational_Accountability on Organizational_Health	0.205	0.121	-0.003	0.413	1.933	0.053
Political_Behavior on Organizational_Accountability	0.633	0.784	0.481	0.784	8.173	0.000
Political_Behavior on Organizational_Health (Direct Effect)	1.051	0.765	0.829	1.272	9.29	< 0.001
Indirect Effect	0.129	0.094	0.000	0.259	1.95	0.050
Total Effect	1.180	0.860	0.984	1.375	11.82	< 0.001

## Discussion

This study aimed to investigate the impact of good governance with the mediating role of organizational accountability on the organizational health of employees in Qazvin hospitals. The results showed that good governance has a positive and significant effect on organizational accountability, consistent with Alfathi [8], stating that good governance based on political and economic stability leads to better accountability. Hospitals need to also use the indicators of good governance, such as attention to the achievement of goals, meritocracy, and prioritizing ethical principles so that they do not face obstacles in being accountable for their performance. Good governance requires that accountability be increased, as a good governor seeks to inform the public of their successful performance. On the other hand, accountability is another dimension of good governance. Governance suggests a combination of the exercise of authority, in which each sector is accountable to the extent of its authority. Additionally, decision-makers in each sector will be accountable and responsible for public issues.

The findings of this study underscore the positive and significant impact of good governance on organizational health, a result consistent with the work of Yaghubi et al. [12], who emphasized good governance as a foundational element for a healthy organizational environment. This relationship is further supported by research across various sectors. For instance, studies in the public sector [13] have demonstrated that transparent and accountable governance practices are strongly associated with improved organizational performance and employee well-being. Similarly, in the healthcare sector, research has shown that organizations with strong governance structures, characterized by effective leadership and clear communication, tend to exhibit higher levels of organizational health, leading to better patient outcomes. These findings suggest that the positive influence of good governance on organizational health is a broad phenomenon, not limited to specific contexts [14].

Furthermore, our results confirm a positive relationship between work experience and organizational health, aligning with the findings of Lee et al. [15], who highlighted the importance of experienced staff in fostering a healthy work environment. This is consistent with research in the technology sector, where found that experienced employees possess a deeper understanding of organizational processes and are better equipped to contribute to a positive and productive work environment [16]. Moreover, a study in the non-profit sector by demonstrated that work experience is a significant factor in improving organizational effectiveness and employee satisfaction, which are closely related to organizational health [17].

Employee experience, encompassing all interactions and touchpoints within the organization, as defined by [18] plays a crucial role in shaping organizational health. It extends beyond tangible factors to include recognition, support, meaningful work, and opportunities for individual contribution. It emphasizes that a positive employee experience is a crucial driver of employee engagement, which in turn contributes significantly to organizational health. The link between employee experience and organizational health is further supported by research on psychological safety which indicates that employees who feel safe and supported are more likely to contribute positively to the organization’s overall well-being [19]. The quality of employee experience significantly influences organizational commitment and job satisfaction. It demonstrated the strong link between positive employee experiences and affective commitment. Therefore, organizations should prioritize creating a positive and supportive employee experience, and future research should further explore the interplay between employee experience, organizational commitment, and job satisfaction [20].

Our study also revealed that shift work patterns significantly impact organizational health, with individuals on fixed shifts exhibiting higher scores compared to those on rotating shifts. This finding is consistent with Lee et al. [15] who reported that physicians and nurses on day shifts experienced better mental health compared to those on night shifts. The detrimental effects of rotating and night shifts on mental health are well-documented potentially affecting various aspects of an individual’s life, including sleep quality, mood, and cognitive function. This is further supported by research in the nursing field, where studies have linked shift work to increased rates of burnout and decreased job satisfaction [21–23]. Given the critical role of healthcare professionals, improving their sleep quality through better shift scheduling and implementing strategies to mitigate the negative effects of shift work is essential for both employee well-being and the quality of patient care. This could include strategies such as implementing more predictable shift patterns, limiting consecutive night shifts, and providing access to resources for sleep management.

Furthermore, our findings indicate that marital status has a significant impact on good governance, organizational health, and organizational accountability, with married individuals exhibiting higher scores. This is in line with the study by Atef et al. [24], which demonstrated that married individuals reported higher job satisfaction and organizational commitment compared to single individuals. Several factors could contribute to this relationship. For instance, married individuals may experience greater social support, which can buffer against work-related stress and contribute to a more positive work experience. Additionally, marriage may be associated with greater financial stability and a sense of personal fulfillment, which can positively impact an individual’s overall well-being and their engagement at work. However, it is important to acknowledge that these are general trends, and individual experiences can vary greatly. Future research should explore the underlying mechanisms that explain the relationship between marital status and organizational outcomes [25].

This research aimed to investigate the mediating role of organizational accountability in the relationship between good governance and organizational health within hospitals. Our findings suggest that good governance alone may not be sufficient to fully realize organizational health; rather, it is through accountability and transparency that the complete benefits of good governance can be observed. This aligns with the concept of “governance for results,” where accountability mechanisms are crucial for translating good governance practices into tangible improvements in organizational performance [26]. Therefore, we recommend that organizations adopt a long-term perspective on good governance, treating it as an ongoing process rather than a temporary goal. Good governance is increasingly recognized as a critical component of effective management, and its principles are being used as a benchmark for evaluating governance across different countries globally [27]. Organizations should strive to continuously improve their governance practices and ensure accountability at all levels to achieve sustainable organizational health and effectiveness.

## Conclusion

Based on the findings of this research, good governance influnces important organizational variables. Overall, this model and the relationships presented between the variables in it can be realized through the utilization of the capacities of the community in the public, private, and civil sectors, as well as by strengthening the organizational capacity, to provide desirable services. Further advance the understanding of organizational health, future research should pursue several avenues. First, a more comprehensive study with a larger sample size, involving multiple provinces and research centers, is recommended to enhance the generalizability of findings. Second, investigations should separately examine and compare the impact of shift work on accountability and organizational health among administrative and clinical staff within hospitals, recognizing potential differences in these groups. Third, the influence of age on organizational health warrants further exploration. Finally, additional research is needed to investigate the specific relationship between work experience and organizational health, building upon our current findings. These proposed avenues will provide a more nuanced and comprehensive understanding of the factors influencing organizational health.

## Data Availability

The datasets used and/or analyzed during the current study available from the corresponding author on reasonable request. The entire dataset is in Farsi language. The Data can be available in English language for the readers and make available from the corresponding author on reasonable request.
